# Activity of EGFR-tyrosine kinase and ALK inhibitors for *EML4–ALK*-rearranged non–small–cell lung cancer harbored coexisting *EGFR* mutation

**DOI:** 10.1186/1471-2407-13-262

**Published:** 2013-05-29

**Authors:** Akihiko Miyanaga, Kumi Shimizu, Rintaro Noro, Masahiro Seike, Kazuhiro Kitamura, Seiji Kosaihira, Yuji Minegishi, Takehito Shukuya, Akinobu Yoshimura, Masashi Kawamoto, Shinichi Tsuchiya, Koichi Hagiwara, Manabu Soda, Kengo Takeuchi, Nobuyuki Yamamoto, Hiroyuki Mano, Yuichi Ishikawa, Akihiko Gemma

**Affiliations:** 1 Department of Pulmonary Medicine and Oncology, Graduate School of Medicine, Nippon Medical School, Tokyo, Japan; 2 Division of Diagnostic Pathology, Nippon Medical School Hospital, Tokyo, Japan; 3 Department of Clinical Pathology, University Hospital, Mizonokuchi, Teikyo University School of Medicine, Kanagawa, Japan; 4 Saitama Medical School Respiratory Organs Internal Medicine, Saitama, Japan; 5 Division of Functional Genomics, Jichi Medical University, Tochigi, Japan; 6 Division of Pathology, The Cancer Institute, Japanese Foundation for Cancer Research, Tokyo, Japan; 7 Division of Thoracic Oncology, Shizuoka Cancer Center, Shizuoka, Japan; 8 Department of Clinical Oncology, Tokyo Medical University Hospital, Tokyo, Japan

**Keywords:** EML4–ALK, *EGFR* mutation, Lung cancer

## Abstract

**Background:**

The *EML4–ALK* (echinoderm microtubule-associated protein-like 4 gene and the anaplastic lymphoma kinase gene) fusion oncogene represents a novel molecular target in a small subset of non–small–cell lung cancers (NSCLCs). The *EML4–ALK* fusion gene occurs generally in NSCLC without mutations in epidermal growth factor receptor *(EGFR)* and *KRAS*.

**Case presentation:**

We report that a case of *EML4–ALK*-positive NSCLC with *EGFR* mutation had a response of stable disease to both an EGFR tyrosine kinase inhibitor (EGFR-TKI) and ALK inhibitor.

**Conclusions:**

We described the first clinical report of a patient with *EML4–ALK*-positive NSCLC with *EGFR* mutation that had a response of stable disease to both single-agent EGFR-TKI and ALK inhibitor. *EML4–ALK* translocation may be associated with resistance to EGFR-TKI, and EGFR signaling may contribute to resistance to ALK inhibitor in *EML4–ALK*-positive NSCLC.

## Background

The *EML4–ALK* (echinoderm microtubule-associated protein-like 4 gene and the anaplastic lymphoma kinase gene) fusion oncogene was recently identified as a novel genetic alteration in non-small-cell lung cancer (NSCLC) [1]. *EML4–ALK* fusions have been detected in 2 to 7% of NSCLC patients. Patients harboring *ALK* rearrangements tend to be never and light smokers, have a history of adenocarcinoma, and be younger in age [1-6]. In general, the *EML4–ALK* fusion oncogene existed exclusively in NSCLC patients without the epidermal growth factor receptor (*EGFR*) gene mutation [1,7,8].

ALK inhibitors such as crizotinib are clinically effective in NSCLC patients harboring ALK rearrangements [9]. Crizotinib produced a high response rate and prolonged median progression-free survival among patients with ALK-positive NSCLC [9]. Crizotinib was recently approved by the US Food and Drug Administration and Japanese Ministry of Health, Labour and Welfare for the treatment of patients with advanced, ALK-rearranged NSCLC.

In this paper, we report a patient with NSCLC with concomitant ALK rearrangement and *EGFR* mutation that had a response of stable disease to both an EGFR tyrosine kinase inhibitor (EGFR-TKI) and ALK inhibitor.

## Case presentation

**Figure 1:**
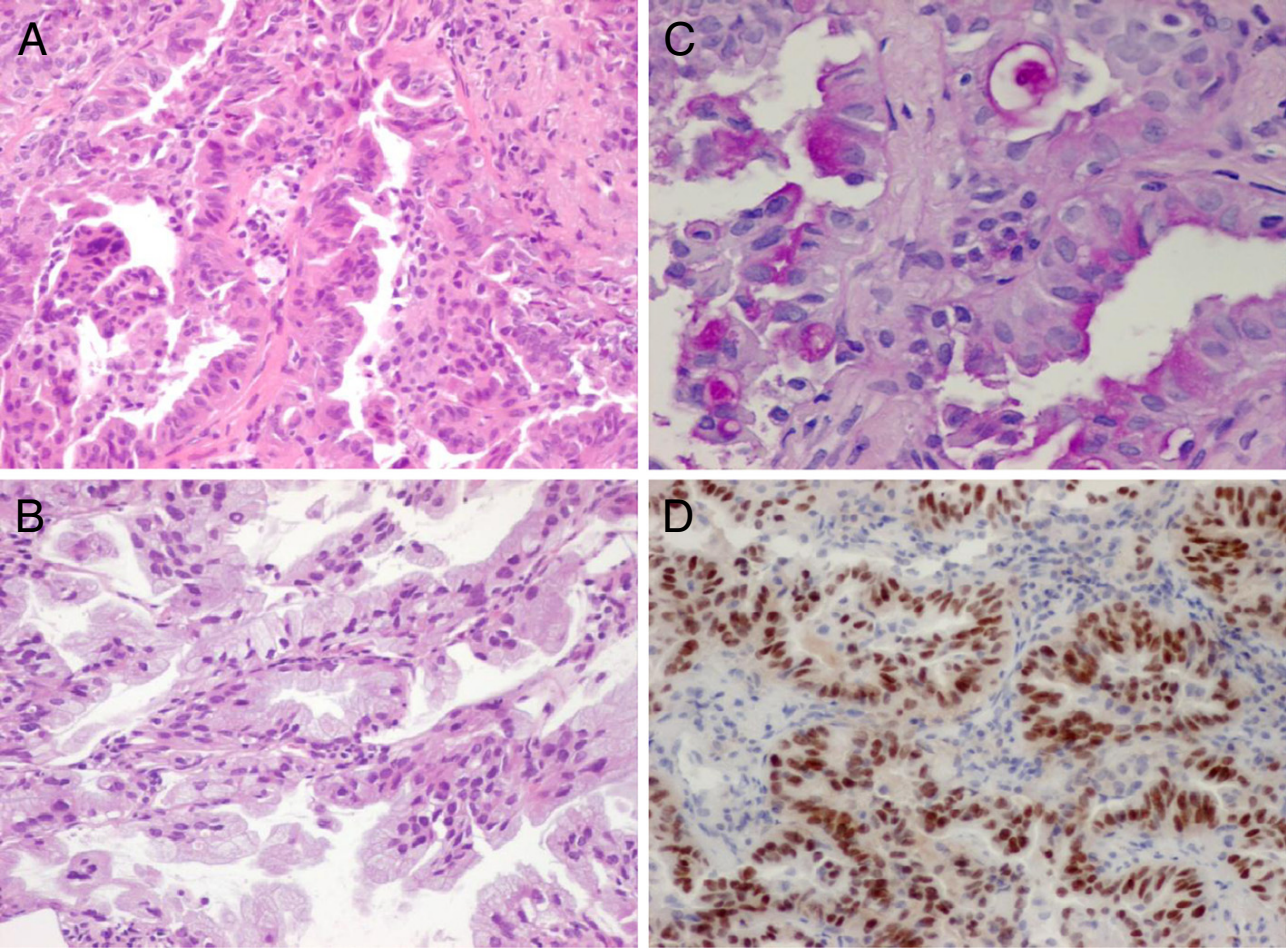
**Histology of the primary tumour.** (**A**) and (**B**) shows a papillary adenocarcinoma (hematoxylin and eosin 200× magnification), (**C**) a mucin stain shows positive for both signet-ring and papillary morphology (PAS, 400× magnification). **(D)** immunohistochemical analysis of lung adenocarcinoma specimens with *EML4-ALK* fusion using a monoclonal anti-TTF-1 antibody (200× magnification).

In December 2009, a 55-year-old female who had never smoked was noted to have left lung opacity on a routine chest X-ray. No significant previous medical history was reported. Computed tomography (CT) scan of the chest revealed a 1.5 × 1.5 cm nodular lesion in the left upper lobe and hilar lymph node metastasis. Transthoracic needle biopsy histology revealed adenocarcinoma, and the histopathological subtype of the specimen was papillary adenocarcinoma with signet-ring cell components (Figure 1A-1C). The specimen was positive for periodic acid–Schiff (PAS) (Figure 1C). On immunohistochemical staining, the tumor cells were positive for thyroid transcription factor-1 (TTF-1) (Figure 1D). Laboratory findings were within normal range, except for the carcinoembryonic antigen (CEA) level of 158.0 ng/mL (normal range, 0 to 4.3 ng/mL) in the serum. She had multiple dorsal vertebra metastases (cT1N1M1b, stage IV).

**Figure 2:**
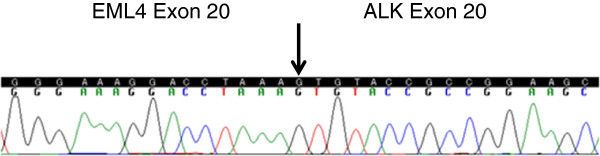
**The sequence of the junction between *EML4 *exon 20 and *ALK *exon 20.**

**Figure 3:**
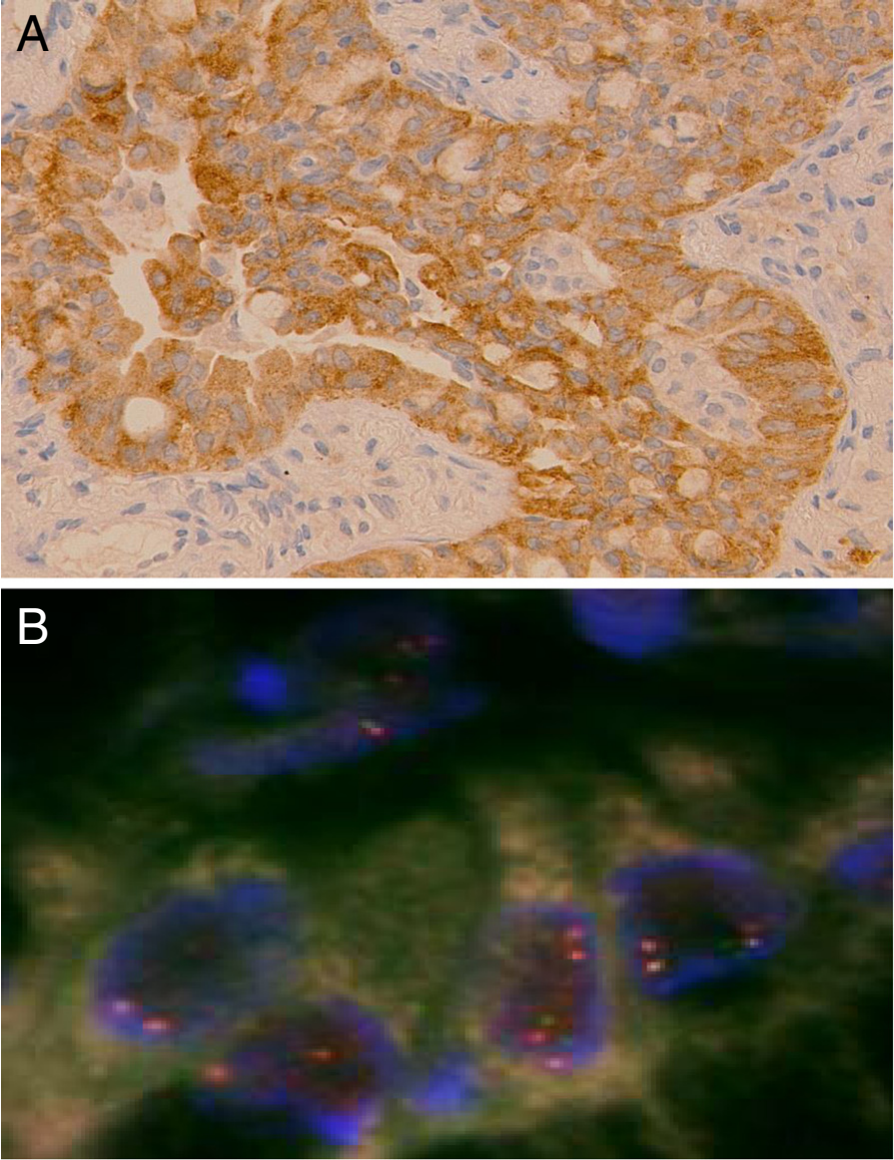
**Diagnosis of an EML4-ALK-positive non-small cell lung cancer.** (**A**) Immunostaining for ALK protein expression in tumor cells. (**B**) The results of a break-apart FISH assay of tumor cells from a patient with rearrangement of the gene encoding *ALK.*

Analysis for *EGFR* gene mutation was performed using a cytological specimen by means of the peptide nucleic acid–locked nucleic acid (PNA-LNA) polymerase-chain-reaction (PCR) clamp method as described previously [10,11]. The specimen showed a deletion in exon 19 (L747-A750del T751S). We collected mRNA from the same tumor specimens using Pinpoint Slide RNA Isolation System in order to clarify whether there was *EML4–ALK* (echinoderm microtubule-associated protein-like 4 gene and the anaplastic lymphoma kinase gene) fusion gene in each tumor. Reverse transcription polymerase-chain-reaction (RT-PCR) followed by direct sequencing confirmed the presence of *EML4–ALK* variant 2 [1] (Figure 2). In addition, *EML4–ALK* was identified by using fluorescent in situ hybridization (FISH) for *ALK* rearrangements (Figure 3B) and was confirmed by immunohistochemistry for ALK expression in tumor [2] (Figure 3A).

A platinum doublet was chosen as first line therapy according to existing treatment protocol in 2009. Four cycles of combination chemotherapy comprising cisplatin and pemetrexed was administered at 3-week intervals. She was judged as having a stable disease. After 7 months, spinal magnetic resonance imaging (MRI) revealed progression of the dorsal vertebra lesions. Therefore, EGFR-TKI was chosen as a 2nd-line therapy. She received gefitinib therapy at 250 mg/day administered orally for 2 months. CT imaging of the chest showed that the pulmonary nodule was not growing after gefitinib therapy, and the tumor marker levels had not changed. However, spinal MRI demonstrated growing dorsal vertebra metastases 2 months after the start of gefitinib therapy. The carcinoembryonic antigen (CEA) level increased from 117 ng/ml to 250 ng/ml. Therefore, the patient was judged as having progressive disease. After local radiation therapy with a total of 30 Gy for dorsal metastases, a second EGFR-TKI was chosen given the stable primary disease. She received another EGFR-TKI, erlotinib (150 mg/day), as 3^rd^-line therapy. After being progression-free for 3 months, spinal MRI revealed a growing thoracic vertebra metastasis. She received 4^th^-line treatment with 2 cycles of docetaxel (DTX). However, her disease progressed 6 months later. Finally, she received a targeted inhibitor of ALK. The patient initially had SD associated with a temporary decrease in the CEA level from 743 ng/ml to 520 ng/ml, but her disease progressed after 4 months of therapy. The ALK inhibitor treatment was ceased and full supportive care was given. All lines of therapy were well tolerated.

## Discussion

We presented a patient with NSCLC with concomitant ALK rearrangement and *EGFR* mutation that had a response of stable disease to both EGFR-TKI and ALK inhibitors. The presence of *EML4–ALK* generally seems to be mutually exclusive of the presence of *EGFR* or *KRAS* mutations in NSCLC [1,7,8]. Previous reports showed twelve cases of *EML4–ALK*-positive lung cancer with *EGFR* mutation [3,12-17]. Only one patient with harboring *ALK* translocation and EGFR mutation was treated by ALK inhibitor has been reported [17]. Lee et al. reported two ALK-positive and EGFR-mutant NSCLC patient who did not respond to EGFR-TKI but achieved a durable partial response to ALK inhibitor [17]. The present patient was a woman with no history of smoking. Her pathological diagnosis was papillary adenocarcinoma with a signet-ring cell component, which was consistent with the previously reported characteristics of *EML4–ALK*-positive lung adenocarcinoma except for the *EGFR* mutation status [1-6]. It was reported that EGFR-TKI therapy among patients with advanced NSCLC and *EGFR* mutations revealed a response rate of more than 60% and progression-free survival of 9 to 14 months [11,18,19]. In addition, recent reports showed that ALK inhibition in NSCLC patients with the *ALK* rearrangement resulted in tumor shrinkage or stable disease in most patients [9]. Unfortunately, EGFR-TKI treatment was not effective in the tumor regression nor tumor marker level of present patient (disease might be controlled), but treatment with an ALK inhibitor resulted in SD with decreasing tumor markers. Therefore, this case showed that ALK rearrangement might be superior to EGFR mutation for the driver mutation.

It was reported that *EML4–ALK* fusion was associated with resistance to EGFR-TKIs [20]. Patients with NSCLC in the *EML4–ALK* cohort and the wild type cohort showed similar response rates to platinum-based combination chemotherapy and no difference in overall survival [20]. Whereas *EGFR* mutations confer sensitivity to EGFR-TKIs, *EML4–ALK* is strongly associated with resistance to EGFR-TKIs. In a previous case of concomitant *EGFR* mutation and *ALK* translocation, the patient presented the most durable response to an EGFR-TKI and was a case demonstrating no EML4*–*ALK expression by immunohistochemistry with an *EML4–ALK* rearrangement characterized by an isolated 3_ FISH signal [12]. Our patient presented a concurrent *EML4-ALK* rearrangement and ALK expression by immunohistochemistry; however, EGFR-TKI was not effective.

Among patients with both *EML4–ALK* rearrangement and *EGFR* mutation, *in vitro* studies showed that EGFR signaling can contribute to ALK inhibitor resistance in EML4*–*ALK NSCLC [14]. In addition, these findings suggested that a cancer cell line that harbors a concurrent *ALK* rearrangement and an *EGFR* mutation would be expected to be resistant to both single agent ALK and EGFR inhibitors [14]. We suggest that the combination of both ALK and EGFR inhibitors as early-line treatment may represent an effective therapy for this subset of NSCLC patients.

## Conclusions

This is the first clinical report of a patient with *EML4–ALK*-positive NSCLC with *EGFR* mutation that had a response of stable disease to both single-agent EGFR-TKI and ALK inhibitor. The *EML4–ALK* fusion gene defines a new molecular subset of NSCLCs with distinct clinical and pathologic features. NSCLCs with *ALK* rearrangement are highly sensitive to ALK inhibition. However, EGFR signaling may contribute to ALK inhibitor resistance in *EML4–ALK* NSCLC. Therefore, we suggest that this provides a translational opportunity whereby laboratory studies should be undertaken to understand the biological link between ALK rearrangement and *EGFR* mutation, with a view to establishing whether there is preclinical justification for using combination therapy for NSCLC with concomitant ALK rearrangement and *EGFR* mutation.

## Consent

Written informed consent was obtained from the patient for publication of this case report and accompanying images.

## Abbreviations

EML4: Echinoderm microtubule-associated protein-like 4; ALK: Anaplastic lymphoma kinase; NSCLC: Non-small cell lung cancer; EGFR: Epidermal growth factor receptor; TKI: Tyrosine kinase inhibitor; CT: Computed tomography; PAS: periodic acid–Schiff; TTF-1: Thyroid transcription factor-1; PNA-LNA: Peptide nucleic acid–locked nucleic acid; PCR: Polymerase chain reaction technique; FISH: Fluorescent in situ hybridization; SD: Stable disease; MRI: Magnetic resonance imaging (MRI); CEA: Carcinoembryonic antigen; RT-PCR: Reverse transcription polymerase chain reaction.

## Competing interests

The authors declare that they have no competing interests.

## Authors’ contributions

AM prepared the manuscript and the literature search; RN and MS reviewed and edited the manuscript; HM and AG corrected and revised the manuscript; KS, KK, SK, YM, MS and TS treated and observed the patient; MK and ST performed the histopathological, immunohistochemical examinations; and AY, KH, KT, NY and YI reviewed the manuscript. All authors read and approved of the final manuscript.

## Pre-publication history

The pre-publication history for this paper can be accessed here:

http://www.biomedcentral.com/1471-2407/13/262/prepub
